# Two Broad Host Range Rhizobial Strains Isolated From Relict Legumes Have Various Complementary Effects on Symbiotic Parameters of Co-inoculated Plants

**DOI:** 10.3389/fmicb.2019.00514

**Published:** 2019-03-15

**Authors:** Vera Safronova, Andrey Belimov, Anna Sazanova, Elizaveta Chirak, Irina Kuznetsova, Evgeny Andronov, Alexander Pinaev, Anna Tsyganova, Elena Seliverstova, Anna Kitaeva, Viktor Tsyganov, Igor Tikhonovich

**Affiliations:** ^1^All-Russia Research Institute for Agricultural Microbiology, Saint Petersburg, Russia; ^2^Kazan Institute of Biochemistry and Biophysics, FRC Kazan Scientific Center, Russian Academy of Sciences, Kazan, Russia; ^3^Sechenov Institute of Evolutionary Physiology and Biochemistry, Russian Academy of Sciences, Saint Petersburg, Russia; ^4^Department of Genetics and Biotechnology, Saint Petersburg State University, Saint Petersburg, Russia

**Keywords:** plant-microbe interaction, symbiosis, nodulation, complementarity of genes, *Astragalus chorinensis*, *Oxytropis popoviana*

## Abstract

Two bacterial strains Ach-343 and Opo-235 were isolated, respectively from nodules of Miocene-Pliocene relict legumes *Astragalus chorinensis* Bunge and *Oxytropis popoviana* Peschkova originated from Buryatia (Baikal Lake region, Russia). For identification of these strains the sequencing of 16S rRNA (*rrs*) gene was used. Strain Opo-235 belonged to the species *Mesorhizobium japonicum*, while the strain Ach-343 was identified as *M. kowhaii* (100 and 99.9% *rrs* similarity with the type strains MAFF 303099^T^ and ICMP 19512^T^, respectively). Symbiotic genes of these strains as well as some genes that promote plant growth (*acdS*, gibberellin- and auxin-synthesis related genes) were searched throughout the whole genome sequences. The sets of plant growth-promoting genes found were almost identical in both strains, whereas the sets of symbiotic genes were different and complemented each other with several *nod*, *nif*, and *fix* genes. Effects of mono- and co-inoculation of *Astragalus sericeocanus*, *Oxytropis caespitosa*, *Glycyrrhiza uralensis*, *Medicago sativa*, and *Trifolium pratense* plants with the strains *M. kowhaii* Ach-343 and *M. japonicum* Opo-235 expressing fluorescent proteins mCherry (red) and EGFP (green) were studied in the gnotobiotic plant nodulation assay. It was shown that both strains had a wide range of host specificity, including species of different legume genera from two tribes (Galegeae and Trifolieae). The effects of co-microsymbionts on plants depended on the plant species and varied from decrease, no effect, to increase in the number of nodules, nitrogen-fixing activity and plant biomass. One of the reasons for this phenomenon may be the discovered complementarity in co-microsymbionts of symbiotic genes responsible for the specific modification of Nod-factors and nitrogenase activity. Localization and co-localization of the strains in nodules was confirmed by the confocal microscopy. Analysis of histological and ultrastructural organization of *A. chorinensis* and *O. popoviana* root nodules was performed. It can be concluded that the strains *M. kowhaii* Ach-343 and *M. japonicum* Opo-235 demonstrate lack of high symbiotic specificity that is characteristic for primitive legume-rhizobia systems. Further study of the root nodule bacteria having complementary sets of symbiotic genes will contribute to clarify the evolutionary paths of legume-rhizobia relationships and the mechanisms of effective integration between partners.

## Introduction

It has been shown that primitive tropical legumes possess relatively low symbiotic specificity, whereas evolutionarily young legumes of temperate climate show higher specificity and can form symbiosis with a single species of root nodule bacteria (Lie et al., 1987; Tikhonovich and Provorov, 2009; Provorov and Vorobyev, 2011; Bakker et al., 2014). The promising models for studying the evolution of such specificity are symbiotic systems of relict legume plants. The species *Vavilovia formosa* growing in Western Asia and Caucasus mountains as well as *Oxytropis triphylla, O. popoviana, O. tragacanthoides, Hedysarum zundukii, Astragalus chorinensis*, and *Glycyrrhiza uralensis* originated from Baikal Lake region are known as Miocene-Pliocene relics (Malyschev, 2006; Sinjushin et al., 2009; Turuta et al., 2015). Information about strains isolated from root nodules of these plant species was recently published (Safronova et al., 2014, 2015a,b, 2017a,b). It was shown that these isolates belonged to different families of rhizobia (*Rhizobiaceae*, *Phyllobacteriaceae*, and *Bradyrhizobiaceae*) and new species *Bosea vaviloviae* and *Phyllobacterium zundukense* were described (Safronova et al., 2015a, 2018b).

The impact of natural rhizobial diversity and the resulting multipartite interactions between symbionts on plant growth is currently poorly understood. On the one hand, it was shown that the multi-strain treatments of two Australian wattle species *Acacia salicina* and *A. stenophylla* interacting with highly diverse communities of rhizobia had a negative effect on plant growth, probably due to strong competition between strains with different levels of nitrogen-fixing activity and symbiotic effectiveness (Barrett et al., 2015). On the other hand, two rhizobial strains *Mesorhizobium japonicum* Opo-242 and *Bradyrhizobium* sp. Opo-243 isolated from the same nodule of a relict legume *O. popoviana* significantly accelerated the root nodule formation on the host plant after a combined inoculation (Safronova et al., 2018a). The whole genome sequence analysis of this pair of strains showed the presence in Opo-243 additional genes *nodPQ, nolK*, and *noeL* involved in the modification of Nod factors (NFs) and affecting the specificity of plant-rhizobia interactions. This report demonstrated that taxonomically different strains forming symbiotic systems with relics can be co-microsymbionts infecting the same nodule and promoting the nodulation process due to complementary sets of symbiotic genes.

The aim of this work was to identify eight new rhizobial strains isolated from nodules of *A. chorinensis* originated from Buryatia (Baikal Lake region, Russia), to select among the *A. chorinensis* and previously obtained *O. popoviana* isolates a pair of strains having the most diverse sets of symbiotic genes, and to study their effects on various legume species (*A. sericeocanus, O. caespitosa, G. uralensis*, *Medicago sativa*, and *Trifolium pratense*) after mono- and co-inoculations. The species *A. sericeocanus, O. caespitosa*, and *G. uralensis* were chosen because they have the same distribution area with *A. chorinensis* and *O. popoviana* plants. The histological and ultrastructural analyzes of *A. chorinensis* and *O. popoviana* root nodules were also conducted to search for specific features of their organization.

## Materials and Methods

### Isolation of Rhizobial Strains From the Root Nodules of *A. chorinensis* and *O. popoviana* Plants in Pot Experiments

Soil samples and seeds of *A. chorinensis* Bunge were collected in Buryatia (Baikal Lake region, Russia). Seeds were surface sterilized and scarified by treatment with 0.1% HgCl_2_ for 10 min and then 5% NaOCl for 8 min, rinsed carefully with sterile tap water and germinated on filter paper in Petri dishes at 25°C in the dark for 4 days. Seedlings were transferred to three sterile plastic pots (5 seedlings per pot) containing 250 g of soil. Plants were cultivated for 60 days in the growth chamber with 50% relative humidity and four-level illumination/temperatures mode: night (dark, 18°C, 8 h), morning (200 μmol m^-2^ s^-1^, 20°C, 2 h), day (400 μmol m^-2^ s^-1^, 23°C, 12 h), evening (200 μmol m^-2^ s^-1^, 20°C, 2 h). Illumination was performed by L 36W/77 FLUORA lamps (Osram, Germany). Then roots of individual plants were removed from soil and washed with tap water. Nodules were analyzed on the stereo microscope Stemi 508 (Carl Zeiss, Germany). Strains of nodule bacteria were isolated from the obtained nodules by the standard method described by Novikova and Safronova (1992) using modified yeast extract mannitol agar (YMA, Vincent, 1970) supplemented with 0.5% succinate (YMSA, Safronova et al., 2015a). From one individual plant one strain was isolated. All strains were deposited in the Russian Collection of Agricultural Microorganisms (RCAM, WDCM 966) and stored at -80°C in the automated Tube Store (Liconic Instruments, Lichtenstein) as described previously (Safronova and Tikhonovich, 2012). Isolation of rhizobial strains from nodules of *O. popoviana* plants originated from the same region as *A. chorinensis* was carried out in our previous work using similar methods (Safronova et al., 2018a).

### Identification of Isolates by the Analysis of the 16S rRNA Gene

For identification of all isolates the following PCR primers were used: fD1 (5′-AGAGTTTGATCCTGGCTCAG -3′) and rD1 (5′-CTTAAGGAGGTGATCCAGCC -3′) for an approximately 1400 bp segment of the 16S rRNA gene (Weisburg et al., 1991). PCR was performed in 25-μL reaction mixtures containing 150 μM dNTPs (Promega, United States), 5 pmol of each primer, 1 U of *Taq* polymerase (Helicon, Russia) and 50–100 ng of purified template DNA. PCR conditions for amplification of the 16S rRNA gene were following: initial denaturation at 95°C for 3 min 30 s; 35 cycles of denaturation at 94°C for 1 min 10 s, annealing at 56°C for 40 s and extension at 72°C for 2 min 10 s; final extension at 72°C for 6 min 10 s. Electrophoresis was carried out with 1% agarose gel (Invitrogen, United States) in TAE. A 100-bp GeneRuler^TM^ and Lambda DNA/HindIII markers (Fermentas, United States) were used for sizing and approximate quantification of DNA fragments. Purification of the PCR products was usually performed by using PureLink^TM^ Quick kit (Invitrogen, United States) according to the manufacturer’s guidance. The direct sequencing of PCR products was performed by an ABI PRISM 3500xl genetic analyzer (Applied Biosystems, United States).

The sequences were compared with related sequences of the type strains available in the GenBank database using BLAST analysis (Basic logical alignment search tool) at NCBI. *Rrs*-dendrogram was constructed using the neighbor-joining method in MEGA 5.0 software package (Tamura et al., 2011). The evolutionary distances were computed using the maximum composite likelihood method. Bootstrap analysis with 1000 replicates was performed to estimate the support of clusters.

All *rrs* sequences have been deposited to the NCBI GenBank database under accession numbers MH626527, MH628053, MH628054, MH628085, MH628088, MH628090 – MH628092.

### Whole Genome Sequencing of the Isolates *M. kowhaii* Ach-343 and *M. japonicum* Opo-235

Genomic DNA was extracted using Genomic DNA Purification KIT (Thermo Fisher Scientific, Europe) according to recommendation of manufacturer. DNA was fragmented by focused ultrasonicator Covaris S2 (Covaris, United States). Fragment DNA-libraries were prepared with NEBNext DNA Library Kit (NEB, United States), and their quality was estimated with High Sensitivity DNA Kit on Bioanalyzer 2100 (Agilent, United States). DNA amount was estimated with dsDNA High Sensitivity Kit on Qubit 1.0 (Invitrogen, United States). Genome sequencing was performed on a MiSeq genomic sequencer (Illumina, United States) by standard protocol with MiSeq Reagent Kit, 600 Cycles (Illumina, United States) at SB RAS Genomics Core Facility (ICBFM SB RAS). Genome was assembled *de novo* using the SPAdes 3.5.0 software (Bankevich et al., 2012). Quality control was performed by QUAST 3.0 (Gurevich et al., 2013). Search for genes in the assembled contigs was performed using the RAST annotation service (Overbeek et al., 2014). Search for homologs of the 16S rRNA gene, ITS region, housekeeping genes *recA*, *glnII*, and *rpoB* as well as symbiotic genes in annotated genomes was performed using CLC Genomics Workbench 7.5.1 software using local BLASTn and tBLASTx.

The whole genome sequences have been deposited to the NCBI GenBank database under accession numbers MZXV00000000 for the isolate *M. kowhaii* Ach-343 and QKOD00000000 for the isolate *M. japonicum* Opo-235. Genomic features of the complete genomes are given in Supplementary Table S1.

### Construction of Fluorescent-Labeled Strains *M. kowhaii* Ach-343 and *M. japonicum* Opo-235

Electroporation of strains was performed in accordance with the previous work (Garg et al., 1999). For this purpose, 90 μl competent cells of strains *M. kowhaii* Ach-343 and *M. japonicum* Opo-235 were suspended between two electrodes spaced by 0.1 cm. Electroporation was carried out with an electric pulse of 14 kV/cm and pulse length of ∼7.3 ms (Gene PulserXcell, Bio-Rad, United States). Number of high voltage pulses ranged 1 for each strain. After the pulse was delivered, the cuvettes were kept on ice for 10 min. Then the electroporated cells were suspended in YM broth (Vincent, 1970), incubated for 24 h at 30°C with 200 rpm shaking (Orbital Shaker-Incubator ES20, BioSan, Latvia) and spreaded on the YMA plates. In transformation experiments a derivative of the pHC60 (tetR) plasmid (Cheng and Walker, 1998), in which the GFP coding sequence was replaced by the mCherry coding sequence (J. Fournier, LIPM, Toulouse, France, unpublished results) and pMP4655 (tetR) plasmid harboring the *egfp* gene were used for electroporation of strains Ach-343 and Opo-235, respectively. Screening of different transformants was performed on YMA medium containing 10 μg/ml tetracycline. PCR was carried out using Thermal Cycler T100 (Bio-Rad, United States) to confirm the transformation using total DNA as template and a pair of primers flanking fluorescent protein gene present on the plasmids pHC60 and pMP4655. The PCR conditions used for the amplification of 1083 and 967 bp fragment (for pHC60 and pMP4655, respectively) included a pre-amplification denaturation at 95°C for 3 min 30 s followed by 35 cycles at 94°C for 1 min 10 s, 54°C for 1 min and 72°C for 2 min 10 s, with a final extension at 72°C for 7 min. The following primers were used: M13F (5′-GTTGTAAAACGACGGCCAGTG-3′) and M13R (5′-AGCGGATAACAATTTCACACAGGA-3′). The PCR products were visualized by electrophoresis on 1.0% agarose gel (Invitrogen, United States) in TAE buffer and purified by using Silica (Helicon, Russia). Sequencing was performed using the ABI PRISM 3500xl (Applied Biosystems, United States) according to the manufacturer’s instructions. Positive clones of each strain were tested for fluorescence in the Axio Imager A1 microscope (Carl Zeiss, Germany).

### Plant Nodulation Assays

Seeds of *A. chorinensis*, *A. sericeocanus*, *O. popoviana*, *O. caespitosa, G. uralensis*, *M. sativa*, and *T. pratense* were surface sterilized, scarified and germinated as described above. The uniformly germinated seedlings were transferred to polypropylene pots OS140BOX (Duchefa, Netherlands) containing 20 g of vermiculite (3 seeds per pot, 4 pots per each treatment of *A. chorinensis* and *O. popoviana* plants, 10 pots per each treatment of other plant species). Each pot was supplemented with 40 ml of the nutrient solution (g/l): K_2_HPO_4_ 1.0, KH_2_PO_4_ 0.25, MgSO_4_ 1.0, Ca_3_(PO_4_)_2_ 0.2, FeSO_4_ 0.02, H_3_BO_3_ 0.005, (NH_4_)_2_MoO_4_ 0.005, ZnSO_4_ × 7 H_2_O 0.005, MnSO_4_ 0.002 (Novikova and Safronova, 1992). Seedlings were inoculated with unlabeled strains *M. kowhaii* Ach-343 and *M. japonicum* Opo-235 or their fluorescent-labeled variants Ach-343(pHC60) and Opo-235(pMP4655) or their combination in the amount of 10^6^ cells per pot. The uninoculated plants were used as negative control. Plants were cultivated for 30 days in the growth chamber as described above. The appearance of nodules on six plants per treatment was recorded after 2 and 3 weeks of cultivation. Nodules were analyzed on the stereo microscope Stemi 508 (Carl Zeiss, Germany) and photos were taken using the microscope color camera AxioCam ERc 5 s (Carl Zeiss, Germany). At the end of experiment the nodules were counted and the fresh biomass of plants (shoots and roots) was determined. The nitrogen fixation of nodules was measured by the acetylene-reduction method (Turner and Gibson, 1980) using gas chromatograph GC-2014 (Shimadzu, Japan). Five nodules from each treatment were used for the confocal microscopy. The data were processed by the standard method of variance analysis using the software STATISTICA version 10 (StatSoft Inc., United States). Fisher’s LSD test was used to evaluate differences between means.

### Confocal Microscopy

Nodules were molded in 3% agarose gel blocks and prepared in 1/4 MTSB (50 mM PIPES, 5 mM MgSO_4_ ⋅ 7H_2_O, 5 mM EGTA, pH 6.9). Nodule sections (50 μm) were prepared using a microtome with a vibrating blade HM650V (Microm, Germany). Sections were analyzed using the laser scanning confocal system LSM 510 META (Carl Zeiss, Germany). EGFP and mCherry were excited at 488 and 543 nm, respectively. Images were acquired with ZEN 2009 software (Carl Zeiss, Germany).

### Histological and Ultrastructural Analysis of *A. chorinensis* and *O. popoviana* Root Nodules Obtained in Gnotobiotic Conditions

The *A. chorinensis* and *O. popoviana* plants inoculated with the strains *M. kowhaii* Ach-343 and *M. japonicum* Opo-235, respectively, were grown in the plant nodulation assay as described above. 3-week-old nodules were harvested from roots and placed directly in fixative. The sample preparation and embedding procedure were described previously (Serova et al., 2018). Briefly, the whole nodules were fixed in 2.5% (v/v) glutaraldehyde (Sigma-Aldrich, United States), then were post-fixed in 2% (*v/v*) osmium tetroxide for 2 h. Samples then were dehydrated in a graded series of increasing ethanol concentrations followed by two changes of 100% acetone. Dehydrated samples were progressively embedded in Epon (Honeywell Fluka^TM^, Thermo Fisher Scientific, United Kingdom) at room temperature. Embedded samples were transferred to blocks in fresh resin and polymerized at 60°C for 48 h.

For transmission electron microscopy, 90–100-nm-thick ultrathin sections were cut using a diamond knife (Diatome, Switzerland) on a Leica EM UC7 ultramicrotome (Leica Microsystems, Germany) and collected on copper/palladium grids coated with 4% (w/v) pyroxylin and carbon. The grids containing the sections were counterstained with 2% (w/v) aqueous uranyl acetate for 1 h followed by lead citrate for 1 min. Ultrathin sections of the selected area were examined using a Tecnai G2 Spirit electron microscope (FEI, the Netherlands) at 80 kV. Electron micrographs were taken with Mega View G2 CCD Camera (Olympus-SIS, Germany).

## Results and Discussion

### Isolation of Rhizobial Strains From the *A. chorinensis* and *O. popoviana* Plants and Their Identification by the Analysis of the 16S rRNA Gene

**FIGURE 1:**
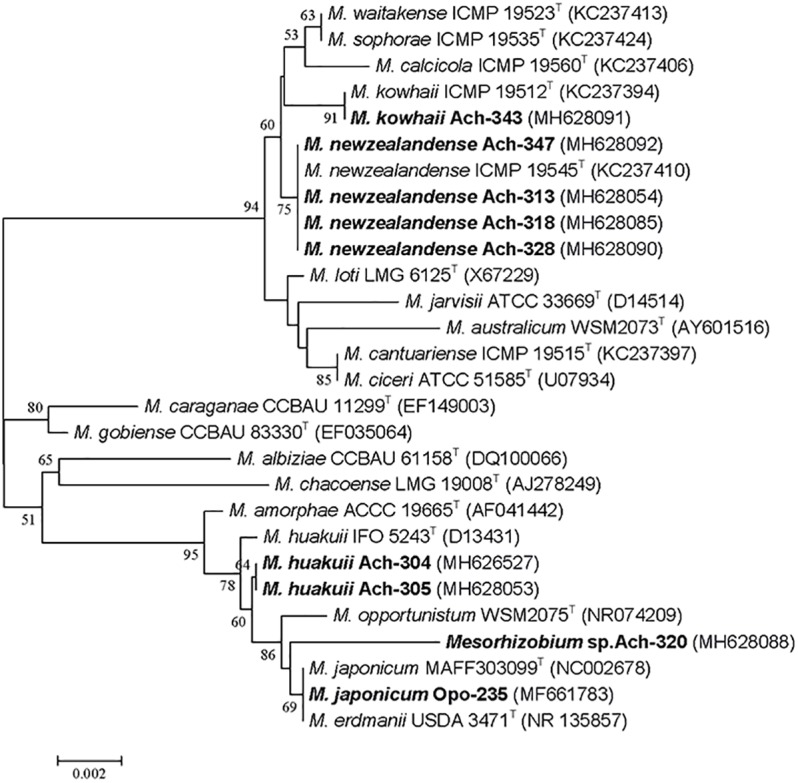
Phylogenetic tree generated by the Neighbor-Joining method using partial 16S rRNA gene sequences (1400 nt) of the *Mesorhizobium*-related isolates from *Astragalus chorinensis* and *Oxytropis popoviana* (in bold) and closely related species. Bootstrap values more than 50% are given. Type strains are indicated by the letter “T.”

Eight fast-growing rhizobial strains (Ach-304, Ach-305, Ach-313, Ach-318, Ach-320, Ach-328, Ach-343 and Ach-347) were isolated from nodules of *A. chorinensis*. Colonies appeared on YMSA medium at the 5-th day. The identification of these isolates by the sequencing of 16S rRNA gene showed that they belonged to different species of the genus *Mesorhizobium* (Figure 1 and Supplementary Table S2). The isolates Ach-313, Ach-318, Ach-328 and Ach-347 formed a separate cluster with the type strain *M. newzealandense* ICMP 19545^T^ (Figure 1) and had 100% *rrs* similarity with this type strain at 94% query cover (Supplementary Table S2). The isolates Ach-304 and Ach-305 grouped together and were the closest to the type strain *M. huakuii* IFO 5243^T^ with 99.93% *rrs* similarity. The isolate Ach-343 formed the high supported cluster with the type strain *M. kowhaii* ICMP 19512^T^ (99.85% *rrs* similarity) while the last isolate Ach-320 was the most closely related to the species *M. japonicum* (99.52% with the type strain MAFF 303099^T^). Thus the isolates Ach-304 and Ach-305 were assigned to the species *M. huakuii* and the isolate Ach-343 to *M. kowhaii.* We preliminarily classified the isolates Ach-313, Ach-318, Ach-328 and Ach-347 as *M. newzealandense*, and left the isolate Ach-320 without species definition. The species *M. newzealandense* and *M. kowhaii* were recently described as microsymbionts of New Zealand endemic species *Sophora prostrata* and *S. microphylla* (De Meyer et al., 2016). The host plant range of these rhizobia is not yet studied; however, the genus *Sophora* includes the extremely promiscuous relict species *S. flavescens* that forms symbiosis with strains belonging to genera *Bradyrhizobium*, *Sinorhizobium*, *Mesorhizobium*, *Rhizobium*, and *Phyllobacterium* (Jiao et al., 2015). On the other hand, the rhizobial species *M. huakuii* being described long ago (Chen et al., 1991) was up to now known as a specific microsymbiont of the plant species *A. sinicus* (Jarvis et al., 1997; Fuli et al., 2017).

Nine other strains belonging to *M. japonicum* and *M. kowhaii* were also isolated in our previous work from the nodules of *O. popoviana* plants (Safronova et al., 2018a). Among them the isolate Opo-235 was chosen as the object of this research and presented in Figure 1, having 100% *rrs* sequence similarity with the type strain *M. japonicum* MAFF 303099^T^ isolated from *Lotus japonicus* (Martínez-Hidalgo et al., 2016).

Thus, eight new isolates from nodules of Miocene-Pliocene relict legume *A. chorinensis* originated from the Baikal Lake region (Eastern Siberia) as well as the previously obtained isolates from *O. popoviana* belonged to mesorhizobial species *M. newzealandense*, *M. huakuii*, *M. kowhaii*, and *M. japonicum* having East Asian or Polynesian origins. Based on whole genome sequence analysis of twelve strains isolated from *O. popoviana*, *O. triphylla*, and *A. chorinensis* plants (data not presented) two strains *M. kowhaii* Ach-343 and *M. japonicum* Opo-235 with the maximum number of symbiotic genes, but with the most different sets of them were selected for further work.

### Analysis of Plant Growth-Promoting and Symbiotic Genes of the Strains *M. kowhaii* Ach-343 and *M. japonicum* Opo-235 by the Whole Genome Sequencing

Symbiotic genes of the strains *M. kowhaii* Ach-343 and *M. japonicum* Opo-235 isolated, respectively, from *A. chorinensis* and *O. popoviana* plants as well as the genes that promote plant growth (*acdS*, gibberellin- and auxin-biosynthesis related genes) were searched throughout the whole genome sequences. It was shown the presence in both strains the *acdS* gene encoded anti-stress enzyme 1-aminocyclopropane-1-carboxylate (ACC) deaminase, which is common among root nodule bacteria (Ma et al., 2003) and plays important role in nodulation process (Glick et al., 2007). The following plant growth-promoting genes were also detected in the strains Ach-343 and Opo-235: *cpxP*, *cpxR*, *cpxU*, and *ispA* related to biosynthesis of phytohormones gibberellins (Nett et al., 2017); the tryptophan synthase (*trp*) and amine oxidase gene involved in the tryptophan-dependent production of auxins (Ahemad and Kibret, 2014). No difference between both strains in the set of plant growth-promoting genes was found, except for the presence of two copies the *acdS* gene in the strain Ach-343 and one copy – in the strain Opo-235 (data not presented).

At the same time, the sets of symbiotic genes of the strains Ach-343 and Opo-235 differed and complemented each other with several *nod*, *nif*, and *fix* genes (Table 1). The common *nodABC* genes necessary for legume nodulation (Wais et al., 2002) were present in both strains. They also contained *nif* and *fix* genes required for nitrogen fixation, namely *nifHDK* and *nifENB* genes encoding structural and catalytic components of the nitrogenase complex (Dos Santos et al., 2012) as well as *fixABCX*, *fixNOPQ*, and *fixGHIS* genes participating in electron transfer to nitrogenase and symbiotically essential oxidase complex (Fischer, 1994; Edgren and Nordlund, 2004). However, the strain Ach-343 had additional *nodG*, *nodM*, *nodN*, and *nifQ* genes that were not found in the strain Opo-235, while the *nodT*, *nodW*, *nifV*, and *fixJKL* genes were observed only in the strain Opo-235. Although the strain Ach-343 contained the protein PZV34926 that has 51% similarity (at 98% coverage) with the NodW protein of the strain *Bradyrhizobium diazoefficiens* USDA 110 (NODW_BRADU) and can play the same role. The functions of these genes and their possible role in the plant-microbe interactions will be discussed below. Bearing in mind that the strains Ach-343 and Opo-235 belong to the microsymbionts of relict rhizobia-legume systems, we calculated the sequence similarity between their *nod*, *nif*, or *fix* genes and homologs sequences presented in the GenBank database. It was shown that 18 *nod* genes of our isolates (*nodCIJGMPQZ* genes of the strain Ach-343 and *nodABCDIJEPWZ* genes of the strain Opo-235) had a high level of similarity (80 - 99%) simultaneously with representatives of two or three rhizobial families: *Rhizobiaceae, Phyllobacteriaceae* and *Bradyrhizobiaceae* (Supplementary Table S3). On the other hand, the *nodABDEFLN* genes of the strain Ach-343 and the *nodFLQT* genes of the strain Opo-235 were the closest only to sequences of strains from the family *Phyllobacteriaceae* (89 – 99% similarity). Phylogenetic trees based on two common nodulation genes *nodA* and *nodC* of isolates and representatives of different rhizobial families (Supplementary Figures S1, S2) showed that both genes involved in biosynthesis of the core structure of NFs (Wais et al., 2002) grouped with other *Mesorhizobium* strains (at 99 and 64% bootstrap support for *nodA* and *nodC*, respectively). The strain *M. amorphae* CCBAU 01583, which is a microsymbiont of *Astragalus membranaceus* and *Caragana intermedia* (Yan et al., 2017), was closest to the isolate Opo-235 (99% of *nodA* and 95% of *nodC* sequence similarity). The isolate Ach-343 revealed close relatedness to the strain *M. septentrionale* CCBAU 11244 isolated from *Caragana microphylla* in China (Chen et al., 2008) with 90% of *nodA* and 94% of *nodC* sequence similarity. It should be noted that the *nodA* gene of the strain Ach-343 was split into two different contigs.

**Table 1:** Presence of symbiotic genes in the strains *Mesorhizobium kowhaii* Ach-343 and *M. japonicum* Opo-235.

Genes	Isolates	
	*M. kowhaii* Ach-343	*M. japonicum* Opo-235
*nod*	*A^∗^BCDIJEF[G]L[MN]PQZ*	*ABCDIJEFLPQ[TW]Z*
*nif*	*ABDEH^∗^KN[Q]STWXZ*	*ABDEHKNST[V]WXZ*
*fix*	*ABCGHINOPQSX*	*ABCGHI[JKL]NOPQSX*
*nol*	–	*[L]*
*noe*	–	*[K]*


*Genes present in only one of strains (Ach-343 or Opo-235) are shown in brackets. Asterisks indicates genes that split into two different contigs.*

### Construction of Fluorescent-Labeled Strains *M. kowhaii* Ach-343 and *M. japonicum* Opo-235

Strains *M. kowhaii* Ach-343 and *M. japonicum* Opo-235 expressing, respectively, fluorescent proteins mCherry (red) and EGFP (green) were obtained using electroporation method. One positive clone of each strain having the brightest fluorescence was selected for further work.

### Symbiotic Phenotype of the Strains *M. kowhaii* Ach-343 and *M. japonicum* Opo-235 in the Plant Nodulation Assays

Symbiotic properties of the unlabeled strains *M. kowhaii* Ach-343 and *M. japonicum* Opo-235 as well as their fluorescent-labeled variants Ach-343(pHC60) and Opo-235(pMP4655) were preliminarily studied in gnotobiotic plant nodulation assay with their host plants *A. chorinensis* and *O. popoviana* (Supplementary Table S4). It was shown, that all strains nodulated both plant species, but formed low effective symbiosis with *O. popoviana* plants (no differences with uninoculated control on the plant biomass). However, they were significantly more effective on *A. chorinensis* plants, although they did not differ in any of the symbiotic parameters. No differences were found between unlabeled strains and their fluorescent-labeled variants (Supplementary Table S4).

**FIGURE 2:**
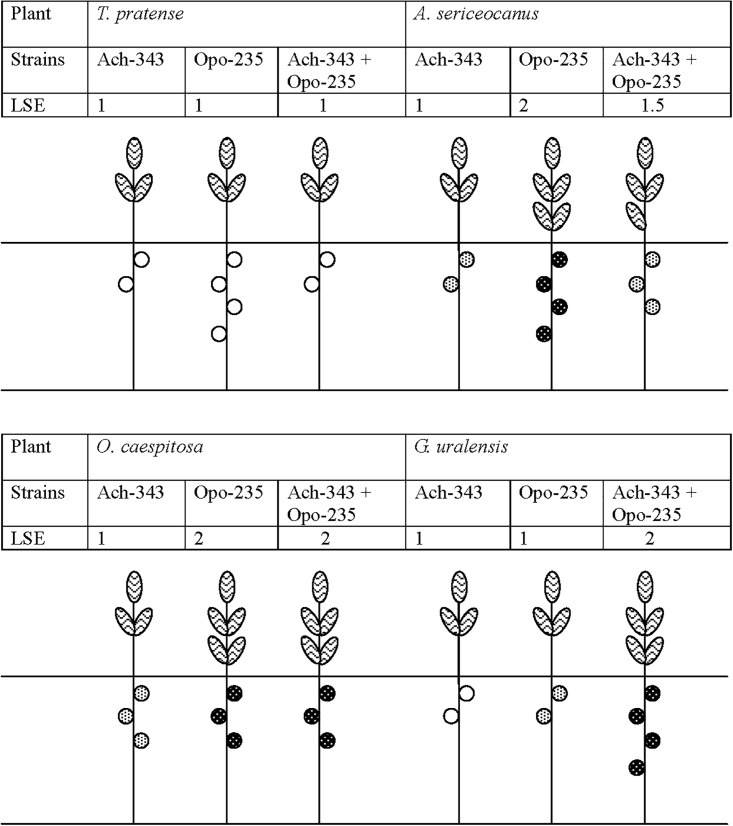
Diagram demonstrating the effects of co-inoculation with strains *M. kowhaii* Ach-343 and *M. japonicum* Opo-235 on the effectiveness of symbioses with *T. pratense, A. sericeocanus, O. caespitosa*, and *G. uralensis* plants. The strains Ach-343 and Opo-235 carried the plasmids pHC60 and pMP4655 and expressed fluorescent proteins mCherry (red) and EGFP (green), respectively. LSE stands for the conditionally level of symbiotic effectiveness (plant biomass). 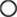 Non-active nodules, 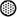 lower level of acetylene reduction activity, 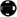 higher level of acetylene reduction activity.

Symbiotic phenotype of the fluorescent-labeled strains *M. kowhaii* Ach-343(pHC60) and *M. japonicum* Opo-235(pMP4655) was studied in gnotobiotic plant nodulation assay with *A. sericeocanus, G. uralensis, O. caespitosa, M. sativa*, and *T. pratense* plants using treatments of mono- and co-inoculation. After 2 weeks of cultivation root nodules were observed only on *A. sericeocanus* roots (in all variants of inoculation) and *G. uralensis* (in the co-inoculation treatment), while after 3 weeks of cultivation only uninoculated controls had no nodules (data not shown). The results of plant nodulation assay (Table 2) demonstrated that the strains Ach-343 and Opo-235 had a wide range of host plants including different species and genera of legumes from tribes Galegeae (*Astragalus, Oxytropis, and Glycyrrhiza*) and Trifolieae (*Medicago, Trifolium*). However, the number of nodules as well as their acetylene reduction activity was different depending on the plant species and treatments: as a rule the strain Opo-235 induced more nodules and was more active in acetylene reduction than the strain Ach-343 (Table 2). Nitrogen-fixing nodules were not observed on *M. sativa* and *T. pratense* plants. The data showed that depending on plant species the effects of a combined inoculation with the strains Ach-343 and Opo-235 on plants varied from the decrease, no effect, to the increase in different symbiotic parameters including plant biomass as an indicator of the symbiotic effectiveness. Figure 2 illustrates this conclusion in the form of a diagram. If the symbiotic effectiveness of the strain Ach-343 is conditionally taken as level 1, the effectiveness of the strain Opo-235 may be taken as levels 1 or 2 (depending on the plant species). With this assumption the symbiotic effectiveness in the variants Ach-343 + Opo-235 can be designated as levels 1, 1.5, or 2, corresponding to the level of one co-microsymbiont (*T. pratense* and *O. caespitosa* plants), to an average level of two strains (*A. sericeocanus* plants) or to the level exceeding both strains (*G. uralensis* plants).

**Table 2:** Effects of mono- and co-inoculation of *Astragalus sericeocanus, Glycyrrhiza uralensis, Oxytropis caespitosa*, *Medicago sativa* and *Trifolium pratense* plants with the strains *M. kowhaii* Ach-343 and *M. japonicum* Opo-235 in the gnotobiotic plant nodulation assay.

Treatment	Host plant												
	*A. sericeocanus*			*G. uralensis*			*O. caespitosa*			*M. sativa*		*T. pratense*	
	NN	PB	ARA	NN	PB	ARA	NN	PB	ARA	NN	PB	NN	PB
Ach-343	4.4 ± 1.4a	235 ± 24ab	14 ± 4a	3.0 ± 0.7a	139 ± 10a	ND	3.3 ± 0.8a	86 ± 8a	10 ± 2a	2.6 ± 0.8a	191 ± 14a	10 ± 3a	169 ± 26a
Opo-235	9.3 ± 2.6b	384 ± 49c	216 ± 78b	1.5 ± 0.6a	159 ± 33a	6 ± 4a	5.0 ± 1.2a	121 ± 13b	31 ± 8b	1.6 ± 0.7a	188 ± 13a	25 ± 12b	189 ± 27a
Ach-343 + Opo-235	5.9 ± 1.3ab	289 ± 16b	32 ± 11a	8.0 ± 1.7b	277 ± 37b	54 ± 38b	3.5 ± 0.4a	113 ± 7b	30 ± 6b	1.9 ± 0.8a	180 ± 15a	11 ± 2a	138 ± 22a
Control without inoculation	ND	190 ± 22a	ND	ND	167 ± 21a	ND	ND	78 ± 14a	ND	ND	170 ± 14a	ND	149 ± 35a


*The strains Ach-343 and Opo-235 carried the plasmids pHC60 and pMP4655 and expressed fluorescent proteins mCherry (red) and EGFP (green), respectively. On *M. sativa* and *T. pratense* plants there were no nitrogen-fixing nodules. The data means ± standard errors of one representative experiment (*n* = 15). Different letters show significant differences between treatments (least significant difference test, *P* < 0.05). NN stands for number of nodules (plant^-*1*^), PB – plant biomass (mg fresh weight plant^-*1*^), ARA stands for acetylene reduction activity (nmol C_*2*_H_*4*_ plant^-*1*^ h^-*1*^), ND stands for not detected.*

### Presence of the Fluorescent-Labeled Variants of Strains *M. kowhaii* Ach-343 and *M. japonicum* Opo-235 in Nodules of *A. sericeocanus, G. uralensis, O. caespitosa, M. sativa*, and *T. pratense*

**FIGURE 3:**
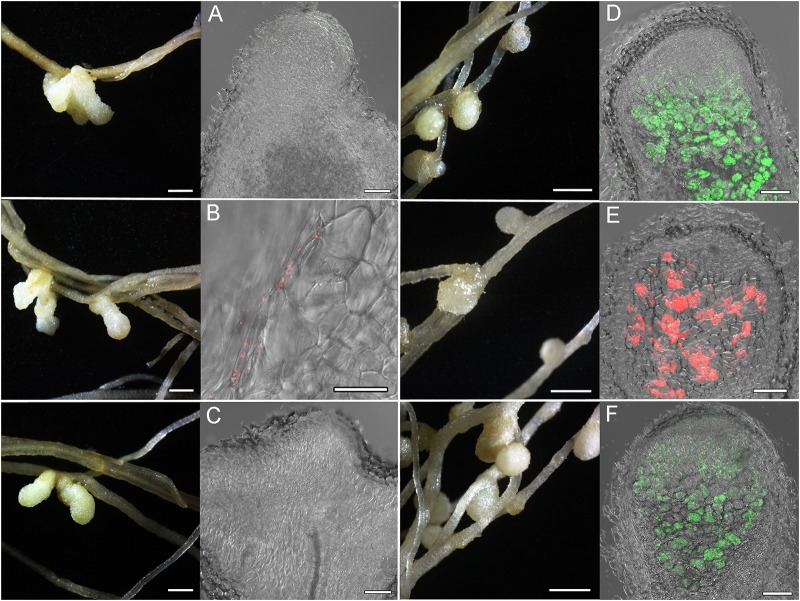
Pictures of root nodules formed by the fluorescent-labeled strains *M. kowhaii* Ach-343(pHC60) and *M. japonicum* Opo-235(pMP4655) on the *M. sativa* and *T. pratense* plants in gnotobiotic nodulation assay. Plant species: **A–C** – *M. sativa*; **D–F** – *T. pratense*. Inoculation treatment: **A,D** – Opo-235, merge of differential interference contrast (DIC) and green channel; **B,E** – Ach-343, merge of DIC and red channel; **C,F** – Opo-235 + Ach-343, merge of DIC, green and red channels. Columns 1 and 3 present the general views of nodules and root surface (scale bar = 1 mm), columns 2 and 4 – the confocal microscopy images (scale bar = 100 μm). Opo-235 bacteria in green, Ach-343 bacteria in red.

**FIGURE 4:**
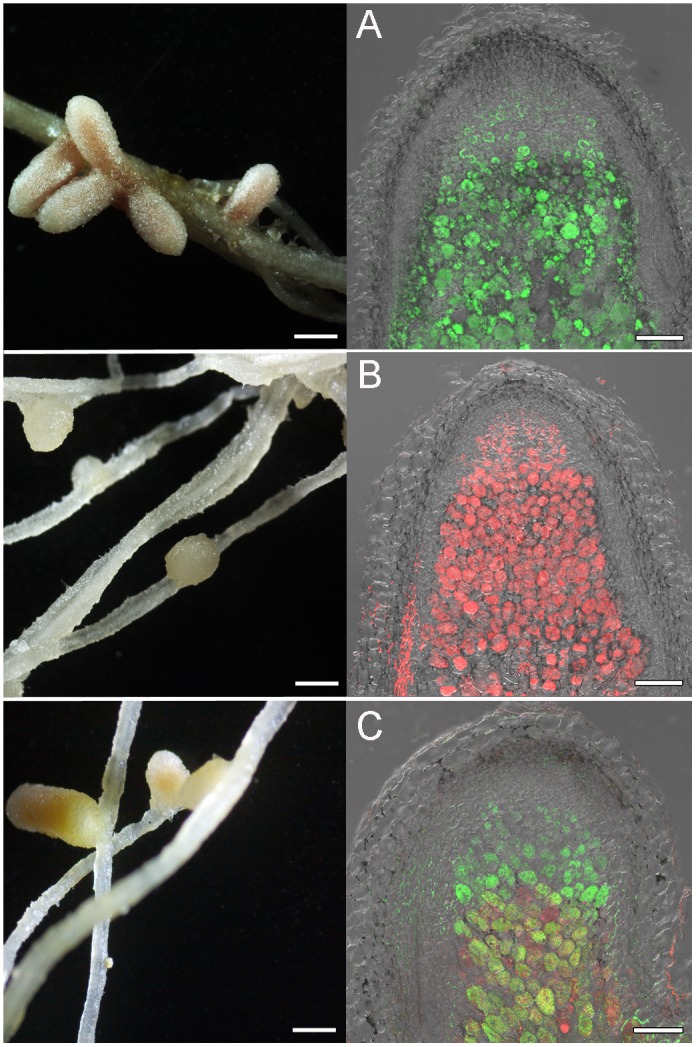
Pictures of root nodules formed by the fluorescent-labeled strains *M. kowhaii* Ach-343(pHC60) and *M. japonicum* Opo-235(pMP4655) on the *A. sericeocanus* and *O. caespitosa* plants in gnotobiotic nodulation assay. Plant species: **A** – *A. sericeocanus*; **B,C** – *O. caespitosa*. Inoculation treatment: **A** – Opo-235 bacteria in green; **B** – Ach-343 bacteria in red; **C** – Opo-235 + Ach-343. Column 1 presents the general views of nodules (scale bar = 1 mm), column 2 – the confocal microscopy images (scale bar = 100 μm). **A** – merge of DIC and green channel; **B** – merge of DIC and red channel; **C** – merge of DIC, green and red channels.

**FIGURE 5:**
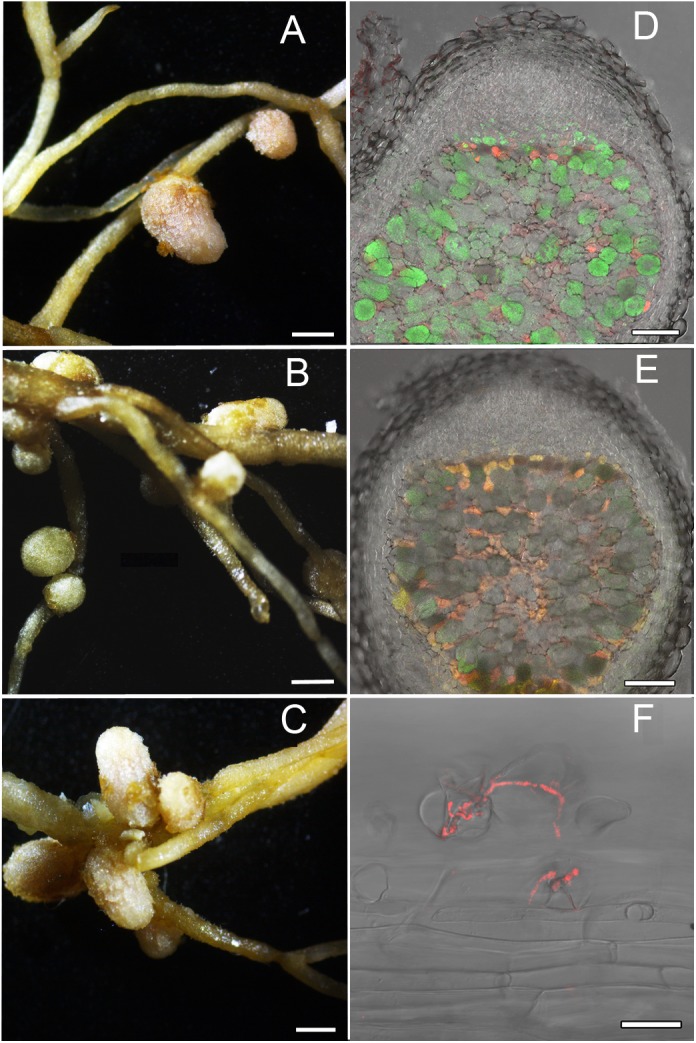
Pictures of root nodules formed by the fluorescent-labeled strains *M. kowhaii* Ach-343(pHC60) and *M. japonicum* Opo-235(pMP4655) on the *G. uralensis* plants in gnotobiotic nodulation assay. Inoculation treatment: **A** – strain Opo-235; **B** – strain Ach-343; **C–F** – Opo-235 + Ach-343; **D,E** – co-infection of plant cells in nodules; **D** – nodule in which the strains occupied the different cells; **E** – nodule in which there are cells with both strains (in yellow-orange); **F** – infection thread formed by the strain Ach-343. Column 1 presents the general views of nodules (scale bar = 1 mm), column 2 – the confocal microscopy images (scale bar = 100 μm). **D,E** – merge of DIC, green and red channels; **F** – merge of DIC and red channel.

Localization and co-localization of the fluorescent-labeled strains *M. kowhaii* Ach-343(pHC60) and *M. japonicum* Opo-235(pMP4655) in nodules obtained in the plant nodulation assay was analyzed by the confocal microscopy (Figure 3–5). Figure 3 represents a general view of nodules on the roots of *M. sativa* and *T. pratense* plants and confocal images of nodule sections. It can be seen that pseudonodules formed on *M. sativa* roots in all variants of inoculation did not contain any rhizobia (Figure 3A–C), although bacterial cells of the strain Ach-343 were present on the root surface (Figure 3B). It is known that *M. sativa* is a highly specific plant that forms a symbiosis only with bacteria *Sinorhizobium meliloti* and *S. medicae*, although it has been shown that some other rhizobial strains can also nodulate this plant species (Noreen et al., 2003; Torres Tejerizo et al., 2011). In contrast, in nodules on *T. pratense* roots inoculated with the strains Opo-235(pMP4655) or Ach-343(pHC60) the corresponding bacteria were present (Figure 3D,E), while in the variant of co-inoculation only nodules with the strain Opo-235(pMP4655) were observed (Figure 3F).

Figure 4, 5 represent some examples of nodules on the roots of *A. sericeocanus, O. caespitosa*, and *G. uralensis* plants. In all variants of mono-inoculation the corresponding strains were detected in nodules. In the co-inoculation treatments all nodules contained the strain Opo-235(pMP4655), however, the strain Ach-343(pHC60) was also observed in some nodules of *O. caespitosa* (Figure 4C) as well as in all nodules and some infection threads of *G. uralensis* (Figure 5D–F). The phenomenon of the simultaneous presence of different rhizobial strains in one nodule, which is called the double nodule occupancy, was previously described for common beans (*Phaseolus vulgaris*) inoculated with the strain *Rhizobium tropici* CIAT 899 marked with the *gus*-gene in competition with other isolates from beans (Shamseldin and Werner, 2004). It should be noted that in nodules of *O. caespitosa* having both strains some plant cells were simultaneously infected by green and red bacteria, but in some plant cells only green or red bacteria were present (Figure 4C). In 3 of the 5 analyzed nodules of *G. uralensis* both strains were predominantly present in the same plant cells (Figure 5E); however, in two nodules the strains occupied different cells and the cells infected by Ach-343 were significantly smaller than the cells infected by Opo-235 (Figure 5D). The observed small size of cells infected with Ach-343 strain may indicate that they do not undergo typical differentiation, which is accompanied by a significant increase in the infected cell size (Tsyganova et al., 2018). However, further studies are required to confirm this assumption. The form of nodules was different depending on the plant species and rhizobial microsymbiont and varied from irregular in shape to round or elongated with one or several lobes (Figure 3–5).

### Histological and Ultrastructural Organization of *A. chorinensis* and *O. popoviana* Root Nodules Formed by the Strains *M. kowhaii* Ach-343 and *M. japonicum* Opo-235

**FIGURE 6:**
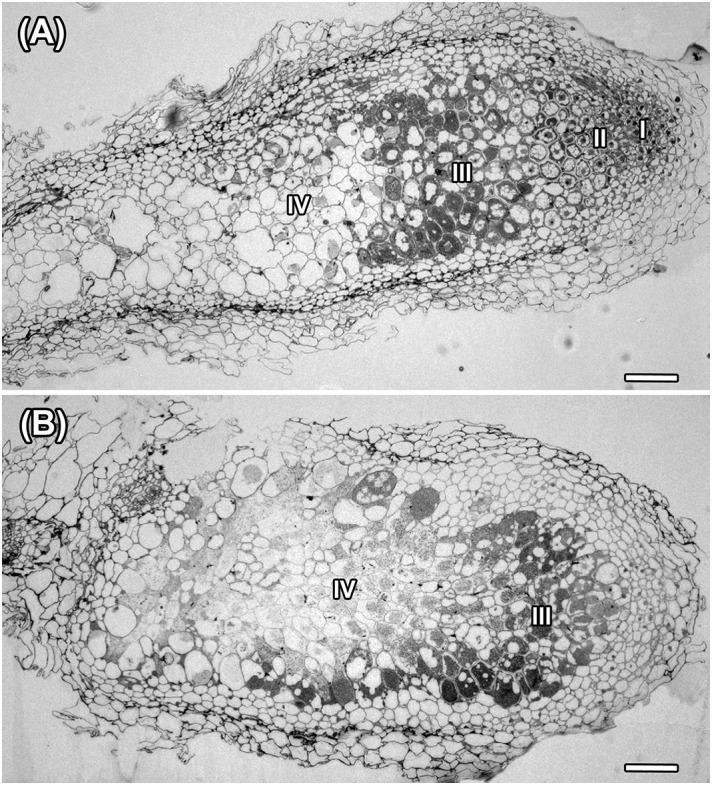
Histological organization in nodules of *A. chorinensis* and *O. popoviana* formed by the strains *M. kowhaii* Ach-343 **(A)** and *M. japonicum* Opo-235 **(B)**. Zones in nodule are designated by Roman numerals: I – meristem, II – infection zone, III – nitrogen fixation zone, IV – senescence zone; scale bar = 100 μm.

**FIGURE 7:**
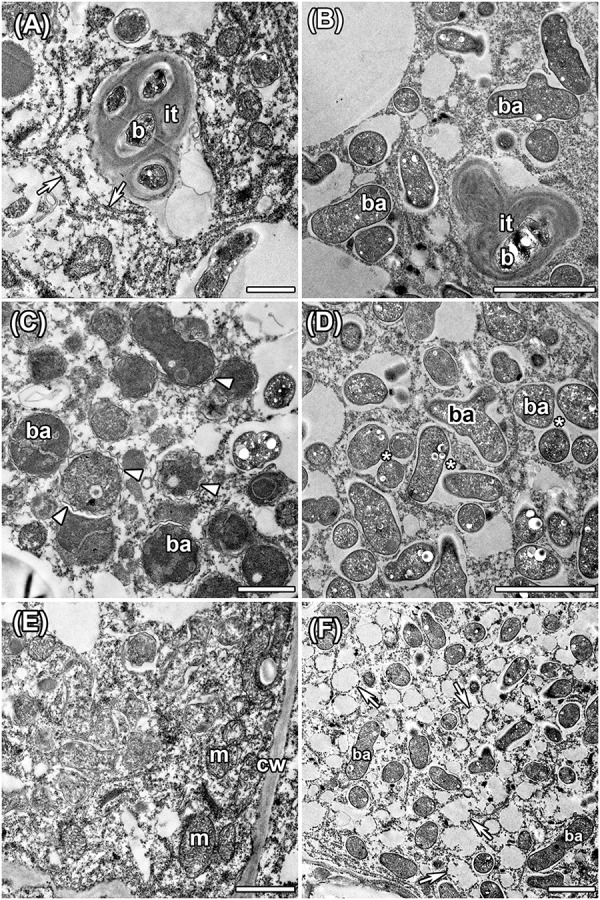
Ultrastructural organization in nodules of *A. chorinensis* and *O. popoviana* formed by the strains *M. kowhaii* Ach-343 **(A,C,E)** and *M. japonicum* Opo-235 **(B,D,F)**. it, infection thread; b, bacterium; ba, bacteroid; m, mitochondria; cw, cell wall; arrowheads indicate rugose symbiosome membrane; arrows indicate endoplasmic reticulum, asterisks indicate symbiosomes with several bacteroids. Scale bars: **(A,C,E)** = 1 μm, **(B,D,F)** = 2 μm.

Histological organization of *A. chorinensis* and *O. popoviana* nodules was typical for indeterminate nodules (Figure 6). Previously, the indeterminate type was described for nodules formed on roots of *A. alpinus*, *O. maydelliana*, *O. arctobia* (Newcomb and Wood, 1986), *A. cicer* (Wdowiak et al., 2000), *A. danicus*, *A. frigidus*, *A. glycyphyllos*, *O. lapponica* (Ampomah et al., 2012). The senescence zone occupied the significant part of nodules due to their large age (Figure 6). The infection threads were well developed in the infection zone in nodules of both species (Figure 7A,B). In nodules of *A. chorinensis* the infected cells were filled by a large number of symbiosomes containing spherical bacteroids individually surrounded with rugose symbiosome membranes (Figure 7C). In *O. popoviana* nodules in infected cells symbiosomes contained several elongated or elongated-branched bacteroids surrounded by a common symbiosome membrane (Figure 7D). In contrast, in *O. maydelliana* nodules bacteroids were surrounded by symbiosome membrane individually (Newcomb and Wood, 1986). Previously, it was shown that in nodules of *A. canadensis* and *O. lamberti* infected cells are filled with elongated bacteroids (Montiel et al., 2017), in nodules of *A. cicer* bacteroids are spherical with elongated outgrowths (Wdowiak et al., 2000) and in nodules of *O. lapponica* bacteroids are elongated-branched (Ampomah et al., 2012). It was suggested that the morphotype of bacteroids correlates with the number of nodule-specific cysteine-rich (NCR) peptides (Montiel et al., 2017). Thus, it is possible that the different morphotypes of bacteroids formed in the nodules of *A. canadensis* and *A. chorinensis* are caused by differences in the compositions of NCR peptides produced by these species. In *A. chorinensis* nodules the numerous mitochondria were present at the periphery of the infected cell (Figure 7E). In nodules of *O. popoviana* in infected cells the endoplasmic reticulum was powerfully developed (Figure 7F). The extensive endoplasmic reticulum was previously described for *A. alpinus*, *O. maydelliana*, *O. arctobia* as an adaptation for nodule development and functioning in arctic conditions (Newcomb and Wood, 1986).

Thus, the strains *M. kowhaii* Ach-343 and *M. japonicum* Opo-235 isolated from nodules of the relict legumes *A. chorinensis* and *O. popoviana*, respectively, had a wide range of host plants, including different genera of legumes (*Astragalus, Oxytropis, Glycyrrhiza, Medicago*, and *Trifolium*). The genetic basis for the low specificity of these isolates may be the similarity of their *nod* genes with representatives of different rhizobial families: *Rhizobiaceae, Phyllobacteriaceae* and *Bradyrhizobiaceae*. This data are in agreement with previous reports suggesting that symbiotic systems of relict plants can be formed with different rhizobial strains belonging to various taxonomic groups (Safronova et al., 2014, 2015a,b, 2017b).

The co-inoculation of plants with both strains Ach-343 and Opo-235, which in some cases were localized in the same nodules, led to changes in symbiotic phenotypes (number of nodules, level of nitrogen fixation and plant biomass) compared with the mono-inoculation treatments. Wherein the effectiveness of symbiosis after co-inoculation could both increase and decrease. This phenomenon of rhizobial interaction can be explained by the complementarity of symbiotic genes, which are responsible for the specific modification of NFs, nitrogenase activity and probably other processes related to formation and development of symbiosis. It was shown that the strains Ach-343 and Opo-235 possess quite different sets of *nod, nif, fix, nol*, and *noe* genes (Table 1). Among them, *nodG*, *nodM*, and *nodN* genes that were observed only in the strain Ach-343 encode the following enzymes that involved in the biosynthesis and modification of NFs: 3-oxoacyl-acyl carrier protein reductase (Lopez-Lara and Geiger, 2001), glucosamine synthase (Baev et al., 1992; Lohrke et al., 1998) and dehydratase (Baev et al., 1992). The strain Opo-235 had two other genes for NFs modification: *noeK* gene participates in NFs fucosylation (Lamrabet et al., 1999), *nolL* gene involved in acetylation of the fucosyl residue (Corvera et al., 1999). The *nodT* and *nodW* genes found only in the strain Opo-235 encode an outer membrane lipoprotein playing a role in the secretion of NFs (Rivilla et al., 1995; Pinto et al., 2009) and the *nod* genes transcription regulator (Perret et al., 2000). It is likely that the differences in the set of *nod*, *nol*, and *noe* genes determine the greater nodule-forming ability of the strain Opo-235 as compared with the strain Ach-343, which was manifested in the plants of *A. sericeocanus* and *T. pratense* (Table 2). It can also be assumed that products of different genes responsible for plant nodulation may interact with each other if they are secreted by bacteria into the external environment or localized on the outer membrane of cells. Acceleration of the root nodule formation has been previously observed in the symbiosis between the relict legume *O. popoviana* and its two co-microsymbionts, one of which could not induce nodules but contained *nodPQ, nolK*, and *noeL* genes that could affect the specificity of plant-rhizobia interactions via the sulfation and fucosylation of NFs (Safronova et al., 2018a).

Particular attention should be given to the observed presence of both strains in the same nodules of *O. caespitosa* and especially *G. uralensis*, where co-microsymbionts were localized in the same plant cells. Along with an increase in the number of nodules, this could be the reason for significant (nine fold) raising the acetylene reduction activity of *G. uralensis* nodules (Table 2). The difference in the specific acetylene reduction activity, calculated on the dry nodule weight, in the variant of co-inoculation was not statistically significant compared to the Opo-235 mono-inoculation treatment due to very large standard errors of this parameter (Supplementary Table S5). However, its increase was 56%. It was shown above that the *nifQ* gene was discovered only in the strain Ach-343, while the *nifV* and *fixJKL* genes were found only in the strain Opo-235 (Table 1). The *nifV* gene is required for a maturation of the nitrogenase MoFe-protein through the homocitrate synthesis (Evans et al., 1991); the *fixJKL* genes are important for expression of some *nif* and *fix* genes (Fischer, 1994). The *nifQ* gene encodes the molybdenum ion binding protein with reductase activity that is known as a specific molybdenum donor for FeMo cofactor biosynthesis and the incorporation of reduced molybdenum in the Mo^IV^ oxidation state into nitrogenase (Hernandez et al., 2009; Black et al., 2012; Boyd et al., 2015). However, the mechanisms of complementation between *nif* and *fix* genes localized in different co-microsymbionts inside the same nodule are not clear. Apparently, the alteration of symbiotic activity may occur due to secreted products of these (or some other) genes, extracellular metabolites or any factors released as a result of bacteroid degradation. One the possible way of such complementation having a positive effect on the nitrogen-fixing activity of symbiosis is an increase in the amount of reduced molybdenum required for high nitrogenase activity and provided by the special Mo-binding proteins.

It should be noted that some other factors besides symbiotic may be involved in the processes of integration between symbiotic partners: plant hormones (auxin, ethylene, gibberellin, cytokinin, abscisic acid), exo- and lipopolysaccharides, cellulases, rhizopines, components of secretion systems (T3SS, T6SS) encoding broad classes of effectors proteins (Russell et al., 2014; Notti and Stebbins, 2016; Okazaki et al., 2016). It was previously shown that the strains *M. kowhaii* Ach-343 and *M. japonicum* Opo-235 did not differ statistically in the activity of the ACC-deaminase reducing the level of the plant stress ethylene and the ability to produce auxins: indole-3-acetic acid (IAA), indole-3-carboxylic acid (ICA), and indole-3-lactic acid (ILA), which are one of the main factors affecting the nodulation process (data not published). The auxin producing activities for the strains Ach-343 and Opo-235 were at the following levels, respectively: 199 and 175 ng/ml for IAA, 111 and 103 ng/ml for ICA, 12 and 15 ng/ml for ILA production. The ACC-deaminase activities of free-living bacteria determined by monitoring the amount of a-ketobutyrate (αKB) generated after hydrolysis of ACC (Saleh and Glick, 2001) by modified method in suspensions of disrupted cells (Belimov et al., 2015) were 14.5 and 12.3 μM αKB mg-1 h-1 for the strains Ach-343 and Opo-235, respectively. However, it should be taken into account that the levels of ACC deaminase activity of mesorhizobia in free-living cells and inside nodules can be different because within this genus the expression of *acdS* genes are usually regulated by NifA protein (Nascimento et al., 2012).

The presence of some components of the T6SS (*icmF, tssABCEGJKL, tagFH, vgrG, vasA, hcp*, and *clpV* genes) was detected in both strains, with the gene sets being identical and the gene similarity between strains was 76 – 81% (data not presented). The genes found are known to encode key components of the bacterial pathogenicity (penetrating system; secretion of cytotoxins, lysozymes, homologs to phage tail proteins, lipoproteins, factors of adherence to epithelial cells). Based on the analysis of secretion systems it remains unclear why the strain Ach-343 demonstrates lower virulence compared with the strain Opo-235 (forms statistically fewer nodules on some plants and is usually not localized inside nodules in the co-inoculation treatments). Further comparative analysis of the factors related to formation and functioning of symbiosis in taxonomic different co-microsymbionts will contribute to the study of the phenomenon of rhizobial interaction, based on genetic complementarity, as well as to the disclosure of the evolutionary paths of legume-rhizobia relationships and the mechanisms of efficient integration between partners.

## Author Contributions

VS and IK participated in microbiology work. AB and AP participated in obtaining of fluorescent-labeled strains. AS and EC participated in molecular work. EA participated in photography of nodules. AT, ES, and VT participated in ultrastructural analysis of root nodules. AK participated in confocal microscopy. IT participated in bio-informatics work.

## Conflict of Interest Statement

The authors declare that the research was conducted in the absence of any commercial or financial relationships that could be construed as a potential conflict of interest.

## References

[B1] Ahemad M. Kibret M. (2014). Mechanisms and applications of plant growth promoting rhizobacteria: current perspective. *J. King Saud. Univ. Sci.* 26 1–20. 10.1016/j.jksus.2013.05.001

[B2] Ampomah O. Y. James E. K. Iannetta P. P. Kenicer G. Sprent J. I. Huss-Danell K. (2012). Nodulation and ecological significance of indigenous legumes in Scotland and Sweden. *Symbiosis* 57 133–148. 10.1007/s13199-012-0188-9

[B3] Baev N. Schultze M. Barlier I. Ha D. C. Virelizier H. Kondorosi E. (1992). *Rhizobium nodM* and *nodN* genes are common *nod* genes: *nodM* encodes functions for efficiency of nod signal production and bacteroid maturation. *J. Bacteriol.* 174 7555–7565. 10.1128/jb.174.23.7555-7565.1992 1447128 PMC207465

[B4] Bakker M. G. Schlatter D. C. Otto-Hanson L. Kinkel L. L. (2014). Diffuse symbioses: roles of plant-plant, plant-microbe and microbe-microbe interactions in structuring the soil microbiome. *Mol. Ecol.* 23 1571–1583. 10.1111/mec.12571 24148029

[B5] Bankevich A. Nurk S. Antipov D. Gurevich A. A. Dvorkin M. Kulikov A. S. (2012). SPAdes: a new genome assembly algorithm and its applications to single-cell sequencing. *J. Comput. Biol.* 19 455–477. 10.1089/cmb.2012.0021 22506599 PMC3342519

[B6] Barrett L. G. Bever J. D. Bissett A. Thrall P. H. (2015). Partner diversity and identity impacts on plant productivity in *Acacia*-rhizobial interactions. *J. Ecol.* 103 130–142. 10.1111/1365-2745.12336

[B7] Belimov A. A. Dodd I. C. Safronova V. I. Shaposhnikov A. I. Azarova T. S. Makarova N. M. (2015). Rhizobacteria that produce auxins and contain 1-amino-cyclopropane-1-carboxylic acid deaminase decrease amino acid concentrations in the rhizosphere and improve growth and yield of well-watered and water-limited potato (*Solanum tuberosum*). *Ann. Appl. Biol.* 167 11–25. 10.1111/aab.12203

[B8] Black M. Moolhuizen P. Chapman B. Barrero R. Howieson J. Hungria M. (2012). The genetics of symbiotic nitrogen fixation: comparative genomics of 14 *Rhizobia* strains by resolution of protein clusters. *Genes* 3 138–166. 10.3390/genes3010138 24704847 PMC3899959

[B9] Boyd E. S. Costas A. M. G. Hamilton T. L. Mus F. Peters J. W. (2015). Evolution of molybdenum nitrogenase during the transition from anaerobic to aerobic metabolism. *J. Bacteriol.* 197 1690–1699. 10.1128/JB.02611-14 25733617 PMC4403663

[B10] Chen W. F. Guan S. H. Zhao C. T. Yan X. R. Man C. X. Wang E. T. (2008). Different *Mesorhizobium* species associated with *Caragana* symbiotic genes and have common host ranges. *FEMS Microbiol. Lett.* 283 203–209. 10.1111/j.1574-6968.2008.01167.x 18422620

[B11] Chen W. X. Li G. S. Qi Y. L. Wang E. T. Yuan H. L. Li J. L. (1991). *Rhizobium huakuii* sp. nov. isolated from the root nodules of *Astragalus sinicus*. *Int. J. Syst. Bacteriol.* 41 275–280. 10.1099/00207713-41-2-275

[B12] Cheng H. P. Walker G. C. (1998). Succinoglycan is required for initiation and elongation of infection threads during nodulation of alfalfa by *Rhizobium meliloti*. *J. Bacteriol.* 180 5183–5191. 9748453 10.1128/jb.180.19.5183-5191.1998 PMC107556

[B13] Corvera A. Promé D. Promé J.-C. Martínez-Romero E. Romero D. (1999). The *nolL* gene from *Rhizobium etli* determines nodulation efficiency by mediating the acetylation of the fucosyl residue in the nodulation factor. *Mol. Plant Microbe Interact.* 12 236–246. 10.1094/MPMI.1999.12.3.236 10065560

[B14] De Meyer S. E. Tan H. W. Andrews M. Heenan P. B. Willems A. (2016). *Mesorhizobium calcicola* sp. nov., *Mesorhizobium waitakense* sp. nov., *Mesorhizobium sophorae* sp. nov., *Mesorhizobium newzealandense* sp. nov. and *Mesorhizobium kowhaii* sp. nov. isolated from *Sophora* root nodules. *Int. J. Syst. Evol. Microbiol.* 66 786–795. 10.1099/ijsem.0.000796 26610329

[B15] Dos Santos P. C. Fang Z. Mason S. W. Setubal J. C. Dixon R. (2012). Distribution of nitrogen fixation and nitrogenase-like sequences amongst microbial genomes. *BMC Genomics* 13:162. 10.1186/1471-2164-13-162 22554235 PMC3464626

[B16] Edgren T. Nordlund S. (2004). The *fixABCX* genes in *Rhodospirillum rubrum* encode a putative membrane complex participating in electron transfer to nitrogenase. *J. Bacteriol.* 186 2052–2060. 10.1128/JB.186.7.2052-2060.2004 15028689 PMC374401

[B17] Evans D. J. Jones R. Woodley P. R. Wilborn J. R. Robson R. L. (1991). Nucleotide sequence and genetic analysis of the *Azotobacter chroococcum* *nifUSVWZM* gene cluster, including a new gene (*nifP*) which encodes a serine acetyltransferase. *J. Bacteriol.* 173 5457–5469. 10.1128/jb.173.17.5457-5469.1991 1885524 PMC208258

[B18] Fischer H.-M. (1994). Genetic regulation of nitrogen fixation in *Rhizobia*. *Microbiol. Rev.* 58 352–386. 7968919 10.1128/mr.58.3.352-386.1994 PMC372973

[B19] Fuli X. Wenlong Z. Xiao W. Jing Z. Baohai H. Zhengzheng Z. (2017). A genome-wide prediction and identification of intergenic small RNAs by comparative analysis in *Mesorhizobium huakuii* 7653R. *Front. Microbiol.* 8:1730. 10.3389/fmicb.2017.01730 28943874 PMC5596092

[B20] Garg B. Dogra R. C. Sharma P. K. (1999). High-efficiency transformation of *Rhizobium leguminosarum* by electroporation. *Appl. Environ. Microbiol.* 65 2802–2804. 10347085 10.1128/aem.65.6.2802-2804.1999 PMC91420

[B21] Glick B. R. Biljana T. Czarny J. Cheng Z. Duan J. McConkey B. (2007). Promotion of plant growth by bacterial ACC deaminase. *Crit. Rev. Plant Sci.* 26 227–242. 10.1080/07352680701572966

[B22] Gurevich A. Saveliev V. Vyahhi N. Tesler G. (2013). QUAST: quality assessment tool for genome assemblies. *Bioinformatics* 29 1072–1075. 10.1093/bioinformatics/btt086 23422339 PMC3624806

[B23] Hernandez J. A. George S. J. Rubio L. M. (2009). Molybdenum trafficking for nitrogen fixation. *Biochemistry* 48 9711–9721. 10.1021/bi901217p 19772354 PMC2999624

[B24] Jarvis B. D. W. Van Berkum P. Chen W. X. Nour S. M. Fernandez M. P. Cleyet-Mare J.-C. (1997). Transfer of *Rhizobium loti*, *Rhizobium huakuii*, *Rhizobium ciceri*, *Rhizobium mediterraneum*, and *Rhizobium tianshanense* to *Mesorhizobium* gen. nov. *Int. J. Syst. Bacteriol.* 47 895–898. 10.1099/00207713-47-3-895

[B25] Jiao Y. S. Liu Y. H. Yan H. Wang E. T. Tian C. F. Chen W. X. (2015). Rhizobial diversity and nodulation characteristics of the extremely promiscuous legume *Sophora flavescens*. *Mol. Plant Microbe Interact.* 28 1338–1352. 10.1094/MPMI-06-15-0141-R 26389798

[B26] Lamrabet Y. Bellogín R. A. Cubo T. Espuny R. Gil A. Krishnan H. B. (1999). Mutation in GDP-fucose synthesis genes of *Sinorhizobium fredii* alters Nod factors and significantly decreases competitiveness to nodulate soybeans. *Mol. Plant Microbe Interact.* 12 207–217. 10.1094/MPMI.1999.12.3.207 10065558

[B27] Lie T. A. Göktan D. Engin M. Pijnenborg J. Anlarsal E. (1987). Co-evolution of the legume-*Rhizobium* association. *Plant Soil* 100 171–181. 10.1007/BF02370940 19909925

[B28] Lohrke S. M. Day B. Kolli V. S. Hancock R. Yuen J. P. de Souza M. L. (1998). The *Bradyrhizobium japonicum* *noeD* gene: a negatively acting, genotype-specific nodulation gene for soybean. *Mol. Plant Microbe Interact.* 11 476–488. 10.1094/MPMI.1998.11.6.476 9612946

[B29] Lopez-Lara I. M. Geiger O. (2001). The nodulation protein NodG shows the enzymatic activity of an 3-oxoacyl-acyl carrier protein reductase. *Mol. Plant Microbe Interact.* 14 349–357. 10.1094/MPMI.2001.14.3.349 11277432

[B30] Ma W. Sebestianova S. B. Sebestian J. Burd G. I. Guinel F. C. Glick B. R. (2003). Prevalence of 1-aminocyclopropane-1-1carboxylate deaminase in *Rhizobium* spp. *Anthony van Leeuwenhoek* 83 285–291. 10.1023/A:1023360919140 12776924

[B31] Malyschev L. I. (2006). *Flora of Siberia, Fabaceae (Leguminosae)*, Vol. 9 Boca Raton, FL: CRC Press 10.1201/b10743

[B32] Martínez-Hidalgo P. Ramírez-Bahena M. H. Flores-Félix J. D. Igual J. M. Sanjuán J. León-Barrios M. (2016). Reclassification of strains MAFF 303099T and R7A into *Mesorhizobium japonicum* sp. nov. *Int. J. Syst. Evol. Microbiol.* 66 4936–4941. 10.1099/ijsem.0.001448 27565417

[B33] Montiel J. Downie J. A. Farkas A. Bihari P. Herczeg R. Bálint B. (2017). Morphotype of bacteroids in different legumes correlates with the number and type of symbiotic NCR peptides. *Proc. Natl. Acad. Sci. U.S.A.* 114 5041–5046. 10.1073/pnas.1704217114 28438996 PMC5441718

[B34] Nascimento F. X. Brígido C. Glick B. R. Oliveira S. (2012). ACC deaminase genes are conserved among *Mesorhizobium* species able to nodulate the same host plant. *FEMS Microbiol. Lett.* 336 26–37. 10.1111/j.1574-6968.2012.02648.x 22846039

[B35] Nett R. S. Montanares M. Marcassa A. Lu X. Nagel R. Charles T. C. (2017). Elucidation of gibberellin biosynthesis in bacteria reveals convergent evolution. *Nat. Chem. Biol.* 13 69–74. 10.1038/nchembio.2232 27842068 PMC5193102

[B36] Newcomb W. Wood S. M. (1986). Fine structure of nitrogen-fixing leguminous root nodules from the Canadian Arctic. *Nord. J. Bot.* 6 609–626. 10.1111/j.1756-1051.1986.tb00461.x

[B37] Noreen S. Schlaman W. R. M. Bellogin R. A. Buendia-Claveria A. M. Espuny M. R. Harteveld M. (2003). Alfalfa nodulation by *Sinorhizobium fredii* does not require sulfated Nod-factors. *Funct. Plant Biol.* 30 1219–1232. 10.1071/FP03093 32689103

[B38] Notti R. Q. Stebbins C. E. (2016). The structure and function of type III secretion systems. *Microbiol. Spectr.* 4 1–18. 10.1128/microbiolspec.VMBF-0004-2015 26999392 PMC4804468

[B39] Novikova N. Safronova V. (1992). Transconjugants of *Agrobacterium radiobacter* harbouring sym genes of *Rhizobium galegae* can form an effective symbiosis with *Medicago sativa*. *FEMS Microbiol. Lett.* 93 261–268. 10.1111/j.1574-6968.1992.tb05107.x 1499987

[B40] Okazaki S. Tittabutr P. Teulet A. Thouin J. Fardoux J. Chaintreuil C. (2016). Rhizobium-legume symbiosis in the absence of Nod factors: two possible scenarios with or without the T3SS. *ISME J.* 10 64–74. 10.1038/ismej.2015.103 26161635 PMC4681849

[B41] Overbeek R. Olson R. Pusch G. D. Olsen G. J. Davis J. J. Disz T. (2014). The SEED and the rapid annotation of microbial genomes using subsystems technology (RAST). *Nucleic Acids Res.* 42 D206–D214. 10.1093/nar/gkt1226 24293654 PMC3965101

[B42] Perret X. Staehelin C. Broughton W. J. (2000). Molecular basis of symbiotic promiscuity. *Microbiol. Mol. Biol. Rev.* 64 180–201. 10.1128/MMBR.64.1.180-201.2000 10704479 PMC98991

[B43] Pinto F. G. Chueire L. M. Vasconcelos A. T. Nicolás M. F. Almeida L. G. Souza R. C. (2009). Novel genes related to nodulation, secretion systems, and surface structures revealed by a genome draft of *Rhizobium tropici* strain PRF 81. *Funct. Integr. Genomics* 9 263–270. 10.1007/s10142-009-0109-z 19184146

[B44] Provorov N. A. Vorobyev N. I. (2011). Evolution of legume-rhizobium symbiosis for an improved ecological efficiency and genotypic specificity of partner interactions. *Genetika* 47 417–424. 21542310

[B45] Rivilla R. Sutton J. M. Downie J. A. (1995). *Rhizobium leguminosarum* NodT is related to a family of outer-membrane transport proteins that includes TolC, PrtF, CyaE and AprF. *Gene* 161 27–31. 10.1016/0378-1119(95)00235-X 7642132

[B46] Russell A. B. Peterson S. B. Mougous J. D. (2014). Type VI secretion system effectors: poisons with a purpose. *Nat. Rev. Microbiol.* 51 584–593. 10.1038/nrmicro3185 24384601 PMC4256078

[B47] Safronova V. Belimov A. Andronov E. Popova J. Tikhomirova N. Orlova O. (2017a). Method for obtaining root nodules of the Baikal relict legumes in laboratory pot experiments. *Int. J. Environ. Stud.* 74 700–705. 10.1080/00207233.2017.1283948

[B48] Safronova V. Belimov A. Sazanova A. Kuznetsova I. Popova J. Andronov E. (2017b). Does the miocene-pliocene relict legume *Oxytropis triphylla* form nitrogen-fixing nodules with a combination of bacterial strains? *Int. J. Environ. Stud.* 74 706–714. 10.1080/00207233.2017.1283947

[B49] Safronova V. Belimov A. Sazanova A. Chirak E. Verkhozina A. Kuznetsova I. (2018a). Taxonomically different co-microsymbionts of a relict legume *Oxytropis popoviana* have complementary sets of symbiotic genes and together increase the efficiency of plant nodulation. *Mol. Plant Microbe Interact.* 31 833–841. 10.1094/MPMI-01-18-0011-R 29498565

[B50] Safronova V. I. Sazanova A. L. Kuznetsova I. G. Belimov A. A. Andronov E. E. Chirak E. R. (2018b). *Phyllobacterium zundukense* sp. nov., a novel species of rhizobia isolated from root nodules of the legume species *Oxytropis triphylla* (Pall.) Pers. *Int. J. Syst. Evol. Microbiol.* 68 1644–1651. 10.1099/ijsem.0.002722 29620492

[B51] Safronova V. Tikhonovich I. (2012). “Automated cryobank of microorganisms: unique possibilities for long-term authorized depositing of commercial microbial strains,” in *Microbes in Applied Research: Current Advances and Challenges*, ed. Mendez-Vilas A. (Hackensack, NJ: World Scientific Publishing Co), 331–334.

[B52] Safronova V. I. Kimeklis A. K. Chizhevskaya E. P. Belimov A. A. Andronov E. E. Pinaev A. G. (2014). Genetic diversity of rhizobia isolated from nodules of the relict species *Vavilovia formosa* (Stev.) Fed. *Antonie van Leeuwenhoek* 105 389–399. 10.1007/s10482-013-0089-9 24292378

[B53] Safronova V. I. Kuznetsova I. G. Sazanova A. L. Kimeklis A. K. Belimov A. A. Andronov E. E. (2015a). *Bosea vaviloviae* sp. nov. a new species of slow-growing rhizobia isolated from nodules of the relict species *Vavilovia formosa* (Stev.) Fed. *Antonie van Leeuwenhoek* 107 911–920. 10.1007/s10482-015-0383-9 25603982

[B54] Safronova V. I. Kuznetsova I. G. Sazanova A. L. Kimeklis A. K. Belimov A. A. Andronov E. E. (2015b). Extra slow-growing *Tardiphaga* strains isolated from nodules of *Vavilovia formosa* (Stev.) Fed. *Arch. Microbiol.* 197 889–898. 10.1007/s00203-015-1122-3 26013968

[B55] Saleh S. S. Glick B. R. (2001). Involvement of *gacS and rpoS* in enhancement of the plant growth-promoting capabilities of *Enterobacter cloacae* CAL2 and UW4. *Can. J. Microbiol.* 47 698–705. 10.1139/w01-072 11575495

[B56] Serova T. A. Tsyganova A. V. Tsyganov V. E. (2018). Early nodule senescence is activated in symbiotic mutants of pea (*Pisum sativum* L.) forming ineffective nodules blocked at different nodule developmental stages. *Protoplasma* 255 1443–1459. 10.1007/s00709-018-1246-9 29616347

[B57] Shamseldin A. Werner D. (2004). Selection of competitive strains of *Rhizobium* nodulating *Phaseolus vulgaris* and adapted to environmental conditions in Egypt, using the gus-reporter gene technique. *World J. Microbiol. Biotechnol.* 20 377–382. 10.1023/B:WIBI.0000033060.27180.8c

[B58] Sinjushin A. A. Demidenko N. V. Gostimskii S. A. (2009). Preliminary report on taxonomical position of *Vavilovia formosa* (Stev.) Fed. morphological evidenced from data molecular. *Pisum Genet.* 41 15–20.

[B59] Tamura K. Peterson D. Peterson N. Stecher G. Nei M. Kumar S. (2011). MEGA5: molecular evolutionary genetics analysis using maximum likelihood, evolutionary distance, and maximum parismony methods. *Mol. Biol. Evol.* 28 2731–2739. 10.1093/molbev/msr121 21546353 PMC3203626

[B60] Tikhonovich I. A. Provorov N. A. (2009). From plant-microbe interactions to symbiogenetics: a universal paradigm for the inter-species genetic integration. *Ann. Appl. Biol.* 154 341–350. 10.1111/j.1744-7348.2008.00306.x

[B61] Torres Tejerizo G. Del Papa M. F. Soria-Diaz M. E. Draghi W. Lozano M. Giusti Mde L. (2011). The nodulation of alfalfa by the acid-tolerant *Rhizobium* sp. strain LPU83 does not require sulfated forms of lipochitooligosaccharide nodulation signals. *J. Bacteriol.* 193 30–39. 10.1128/JB.01009-10 20971905 PMC3019937

[B62] Tsyganova A. V. Kitaeva A. B. Tsyganov V. E. (2018). Cell differentiation in nitrogen-fixing nodules hosting symbiosomes. *Funct. Plant Biol.* 45 47–57. 10.1071/FP16377 32291020

[B63] Turner G. L. Gibson A. H. (1980). “Measurement of nitrogen fixation by indirect means,” in *Methods for Evaluating Biological Nitrogen Fixation*, ed. Bergensen F. J. (Toronto: Wiley), 111–138.

[B64] Turuta O. Ryabtsev V. Novitskaya N. Vakarenko L. (2015). *Protected Microhabitats as a Part of Baikal Regional Ecological Network. Plant Microreserves the Site Based Plant Conservation And Monitoring Network.* Available at: http://cretaplant.biol.uoa.gr/docs/Turuta2001.pdf

[B65] Vincent J. M. (1970). *A Manual for the Practical Study of Root Nodule Bacteria in IBP Handbook.* Oxford: Blackwell Scientific Publications, 73–97.

[B66] Wais R. J. Keating D. H. Long S. R. (2002). Structure-function analysis of Nod factor-induced root hair calcium spiking in *Rhizobium*-legume symbiosis. *Plant Physiol.* 129 211–224. 10.1104/pp.010690 12011352 PMC155885

[B67] Wdowiak S. Małek W. Sajnaga E. Łotocka B. Stepkowski T. Legocki A. (2000). Symbiosis of *Astragalus cicer* with its microsymbionts: partial *nodC* gene sequence, host plant specificity, and root nodule structure. *Antonie Van Leeuwenhoek* 78 63–71. 10.1023/A:1002738609114 11016697

[B68] Weisburg W. G. Barns S. M. Pelletier D. A. Lane D. J. (1991). 16S ribosomal DNA amplification for phylogenetic study. *J. Bacteriol.* 173 697–703. 10.1128/jb.173.2.697-703.1991 1987160 PMC207061

[B69] Yan H. Xie J. B. Ji Z. J. Yuan N. Tian C. F. Ji S. K. (2017). Evolutionarily conserved *nodE, nodO*, T1SS, and hydrogenase system in rhizobia of *Astragalus membranaceus* and *Caragana intermedia*. *Front. Microbiol.* 8:2282. 10.3389/fmicb.2017.02282 29209294 PMC5702008

