# Supplementary material for: Two Broad Host Range Rhizobial Strains Isolated From Relict Legumes Have Various Complementary Effects on Symbiotic Parameters of Co-inoculated Plants
Source: Front Microbiol. 2019 Mar 15;10:514. doi: 10.3389/fmicb.2019.00514 (PMC6428766; doi:10.3389/fmicb.2019.00514)
Supplement: Supplementary file 1 [file Data_Sheet_1.docx]

Supplementary Material

Two Broad Host Range Rhizobial Strains Isolated from Relict Legumes Have Various Complementary Effects on Symbiotic Parameters of Co-inoculated Plants

Vera Safronova^*^, Andrey Belimov, Anna Sazanova, Elizaveta Chirak, Irina Kuznetsova, Evgeny Andronov, Alexander Pinaev, Anna Tsyganova, Elena Seliverstova, Anna Kitaeva, Viktor Tsyganov, Igor Tikhonovich

*** Correspondence:**Dr. Vera Safronova
v.safronova@rambler.ru

**Supplementary Table 1**. **Genomic features** of the isolates *M. kowhaii* Ach-343 and *M. japonicum* Opo-235 **complete genomes.**

| **Genomic feature** | Isolates | |
| --- | --- | --- |
|  | *M. kowhaii* Ach-343 | *M. japonicum* Opo-235 |
| Coverage | 14.0x | 58.0x |
| genome size | 8 062 617 | 7 357 801 |
| GC% | 62.3% | 62.6% |
| CDSs | 7859 | 7222 |

**Supplementary Table 2**. *Rrs* sequence similarities between *Mesorhizobium*-related isolates from *A. chorinensis* and the reference type strains. 1 – *M. huakuii* IFO 5243^T^, 2 – *M. newzealandense* ICMP 19545^T^, 3 *- M. japonicum* MAFF 303099^T^, 4 - *M. kowhaii* ICMP 19512^T^.

| Isolates | *Rrs* sequence similarities (%) | | | |
| --- | --- | --- | --- | --- |
|  | 1 | 2 | 3 | 4 |
| Ach-304  Ach-305  Ach-313  Ach-318  Ach-320  Ach-328  Ach-343  Ach-347 | 99.93  99.93  98.15  98.06  98.82  98.12  97.71  98.13 | 98.26  98.19  100  100  97.79  100  99.62  100 | 99.85  99.86  98.22  98.13  99.52  98.19  97.92  98.20 | 98.21  98.15  99.70  99.70  97.67  99.78  99.85  99.70 |

**Supplementary Table 3**. Sequence similarity of *nod* genes of the isolates *M. kowhaii* Ach-343 and *M. japonicum* Opo-235 with representatives of different rhizobial families: *Rhizobiaceae, Phyllobacteriaceae* and *Bradyrhizobiaceae.* Similarity levels greater than 80% in bold.

| *Nod* genes of the isolates | Sequence similarities with representatives of the rhizobial families (%) | | |
| --- | --- | --- | --- |
|  | *Rhizobiaceae* | *Phyllobacteriaceae* | *Bradyrhizobiaceae* |
| *M. kowhaii* Ach-343 | | | |
| *nodA* | <70 | 90 | 79 |
| *nodB* | 77 | 95 | 75 |
| *nodC* | **81** | **94** | **81** |
| *nodD* | 79 | 89 | 78 |
| *nodG* | **96** | **93** | 72 |
| *nodI* | **88** | **86** | **82** |
| *nodJ* | **88** | **81** | 78 |
| *nodE* | 76 | 92 | 67 |
| *nodF* | 73 | 96 | 72 |
| *nodL* | 73 | 91 | 71 |
| *nodM* | **83** | **99** | 71 |
| *nodN* | 72 | 96 | 71 |
| *nodP* | **94** | **94** | 75 |
| *nodQ* | **93** | **92** | 71 |
| *nodZ* | **80** | **81** | **86** |
| *M. japonicum* Opo-235 | | | |
| *nodA* | **89** | **99** | <70 |
| *nodB* | **80** | **83** | **82** |
| *nodC* | **81** | **95** | **83** |
| *nodD* | **80** | **99** | 79 |
| *nodI* | **82** | **99** | **83** |
| *nodJ* | <70 | **99** | **81** |
| *nodE* | **87** | **99** | <70 |
| *nodF* | 79 | 99 | <70 |
| *nodL* | 77 | 99 | <70 |
| *nodP* | **81** | **99** | 79 |
| *nodQ* | 74 | 99 | 75 |
| *nodT* | <70 | 99 | <70 |
| *nodW* | **88** | **94** | <70 |
| *nodZ* | <70 | **99** | **86** |

**Supplementary Figure 1.** Phylogenetic tree generated by the Neighbour-Joining method using *nodA* gene sequences of the isolates *M. kowhaii* Ach-343 and *M. japonicum* Opo-235 (in bold) with representatives of different rhizobial families: *Rhizobiaceae, Phyllobacteriaceae* and *Bradyrhizobiaceae.*

*M. opportunistum* WSM2075 (CP002279)

*M. australicum* WSM2073 (CP003358)

*M. metallidurans* STM 2683 (CAUM01000171)

*M. wenxiniae* WYCCWR 10195 (NPKH01000001)

*M. muleiense* CGMCC 1.11022 (FNEE01000043)

***M. kowhaii* Ach-343** (MZXV01000084)

*M. septentrionale* CCBAU 11244 (GQ167253)

***M. japonicum* Opo-235** (QKOD01000010)

*M. amorphae* CCBAU 01583 (JHUP01000091)

*S. fredii* NGR234 (NC000914)

*R. etli* CIAT 652 (CP001076)

*B. elkanii* USDA 76 (KB900701)

*B. diazoefficiens* USDA 110 (NC004463)

*B. japonicum* USDA 6 (NC017249)

*S. meliloti* 1021 (NC003037)

*R. leguminosarum* 3841 (NC008381)

100

100

100

100

100

100

97

100

99

85

99

48

99

0.05

**Supplementary Figure 2.** Phylogenetic tree generated by the Neighbour-Joining method using *nodC* gene sequences of the isolates *M. kowhaii* Ach-343 and *M. japonicum* Opo-235 (in bold) with representatives of different rhizobial families: *Rhizobiaceae, Phyllobacteriaceae* and *Bradyrhizobiaceae.*

*M. australicum* WSM2073 (CP003358)

*M. opportunistum* WSM2075 (CP002279)

*M. metallidurans* STM 2683 (CAUM01000171)

*M. metallidurans* STM 2683 (CAUM01000171)

*M. wenxiniae* WYCCWR 10195 (NPKH01000001)

***M. kowhaii* Ach-343** (MZXV01000084)

***M. japonicum* Opo-235** (QKOD01000010)

*M. amorphae* CCBAU 01583 (JHUP01000091)

*M. septentrionale* CCBAU 11244 (GQ167253)

*R. etli* CIAT 652 (CP001076)

*B. elkanii* USDA 76 (KB900701)

*B. japonicum* USDA 6 (NC017249)

*B. diazoefficiens* USDA 110 (NC004463)

*S. fredii* NGR234 (NC000914)

*R. leguminosarum* 3841 (NC008381)

*S. meliloti* 1021 (NC003037)

100

100

100

98

99

100

99

95

70

99

81

72

64

0.1

**Supplementary Table 4**. Symbiotic properties of the unlabeled strains *M. kowhaii* Ach-343 and *M. japonicum* Opo-235 as well as their fluorescent-labeled variants Ach-343(pHC60) and Opo-235(pMP4655) in gnotobiotic plant nodulation assay with their host plants *A. chorinensis* and *O. popoviana.*

The strains Ach-343 and Opo-235 carried the plasmids рHC60 and pMP4655 and expressed fluorescent proteins mCherry (red) and EGFP (green), respectively. The data means ± standard errors of one representative experiment (n = 12). Different letters show significant differences between treatments (least significant difference test, *P* < 0.05). NN stands for number of nodules (plant^-1^), PB - plant biomass (mg fresh weight plant^-1^), ARA stands for acetylene reduction activity (nmol C_2_H_4_ plant^-1^ h^-1^), ND stands for not detected.

| Treatment | Host plant | | | | | |
| --- | --- | --- | --- | --- | --- | --- |
|  | *A. chorinensis* | | | *O. popoviana* | | |
|  | NN | PB | ARA | NN | PB | ARA |
| Ach-343 | 2.7 ± 0.7a | 121 ± 11a | 68 ± 23a | 1.0 ± 0.5a | 68 ± 7a | 20 ± 6a |
| Ach-343(pHC60) | 3.2 ± 0.5a | 142 ± 17a | 76 ± 22a | 1,2 ± 0.7a | 60 ± 7a | 22 ± 7a |
| Opo-235 | 2.8 ± 0.6a | 146 ± 19a | 55 ± 16a | 1.6 ± 0.6a | 73 ± 9a | 16 ± 4a |
| Opo-235(pMP4655) | 2.5 ± 0.7a | 135 ± 18a | 70 ± 19a | 2.0 ± 0.9a | 65 ± 6a | 25 ± 9a |
| Control without inoculation | ND | 76 ± 6b | ND | ND | 65 ± 8a | ND |

**Supplementary Table 5.** Effects of mono- and co-inoculation of *A. sericeocanus, G. uralensis* and *O. caespitosa* plants with the strains *M. kowhaii* Ach-343(pHC60) and *M. japonicum* Opo-235(pMP4655) on the specific acetylene reduction activity (nmol C_2_H_4_ plant^-1^ h^-1^ mg of dry nodule weight^-1^) in the gnotobiotic plant nodulation assay. The data means ± standard errors of one representative experiment (n = 10). Different letters show significant differences between treatments (least significant difference test, *P* < 0.05). ND stands for not detected.

| Treatment | Specific acetylene reduction activity (nmol C_2_H_4_ plant^-1^ h^-1^ mg of dry nodule weight^-1^) on the host plants | | |
| --- | --- | --- | --- |
|  | *A. sericeocanus* | *G. uralensis* | *O. caespitosa* |
| Ach-343(pHC60) | 3 ± 0.6a | ND | 6 ± 1.4a |
| Opo-235(pMP4655) | 15 ± 5.2b | 9 ± 1.9a | 13 ± 2.7b |
| Ach-343(pHC60)+ Opo-235(pMP4655) | 13 ± 2.4b | 14 ± 4.3a | 16 ±2.4b |
